# Network based models for biological applications

**Published:** 2009-04-25

**Authors:** Dobrescu Radu, Purcărea Victor

**Affiliations:** * Polytechnic University of Bucharest, Faculty of Automatic Control and Computers; ** ”Carol Davila” University of Medicine and Pharmacy, Bucharest, Romania

**Keywords:** network models, preferential attachment, tumor growth, food web, scale free graph

## Abstract

This paper analyses the adequacy of different types of networks in biological process modeling. The assumptions are sustained by two case studies. The first one is a lattice-based computer model to simulate the growth of nonvascular tumors with nutrient consumption constraints. The modeling solution is able to reproduce the classic three-layer structure familiar from multicellular spheroids: cell proliferation, quiescent and necrosis. The accuracy of this model is tested by comparing it to a fractal morphometric technique of two patterns, one of them obtained by simulation, the other developed *in vitro*. The second application is the growth of a directed network, in which the growth is constrained by the cost of adding links to the existing nodes. This is a new preferential attachment scheme, different from those specific for the construction of scale-free graphs, because its new nodes prefer to attach to existing nodes with lower degree. We relate this mechanism to a simple food-web model studied by simulations.

## Network models as tools to study complexity

Many processes in natural or artificial systems take place in a discrete space, in which several agents are tangled together in a web of interactions with varying range. The internet, ecosystems, metabolic reactions, social systems, and financial markets are just a few examples. The network concept has proved to be very useful, particularly in studying such complex systems. We do not have a general theory of complexity yet, but only a collection of theories inherited from physics and mathematics, as well as some paradigms and tools, which were developed relatively recently. Today, we can explain some complex phenomena, such as the collective behavior seen in ferromagnetic phase transitions [**1**], herding behavior [**2**], or opinion formation [**3**], in which short-range interactions create long-range order. We also know that even very simple systems, such as the discrete logistic growth model (logistic map), can display very rich and complicated, i.e., chaotic, dynamics [**4**]. Self-organized criticality, to some extent, explains how some systems can manage to operate near criticality without fine-tuning [**5**]. Fractal geometry helps us understand how and why certain forms and structures in nature, such as vascular systems, arise [**6**]. Network theory is another tool used to study complex phenomena, which is built on graph theory, and, in a broader perspective, dynamical systems theory. Generally, problems related to networks can be classified into two categories:

1. Problems related to the topology of the network or its evolution: tolerance attack of communication networks and the spreading of information or diseases in the social or the communicational environment.

2. Problems related to the dynamics on networks: stability of ecosystems, gene regulation, and stock-market fluctuations.

In this paper, we only take into consideration the first kind of problems.

## Networks Topology

A network is a set of nodes (vertices) connected with links (edges). The links can be directed or undirected. The number of links connected to a node is called the degree (connectivity) of that node. If the network is directed, one can also define the respective quantities for incoming and outgoing links, the indegree and outdegree. The degree distribution is one of the main features of a network. An exact definition of a network can be given by an *N × N* adjacency matrix, where *N* is the total number of nodes and each nonzero matrix element, *mij,* , represents a link from node *i* to node *j*. The distance between two nodes is the shortest path between them. The maximum distance between any pair of nodes in the network is called its diameter. The in-between of a node, *i*, is the total number of shortest paths between all possible pairs in the network that pass through *i*. The clustering coefficient is defined as the probability of finding two connected nearest neighbors of a node, averaged over all nodes in the network [**7**].

**Regular Lattices**

Regular lattices are the simplest networks in nature. The most common, if not the only example from nature is crystals, in which atoms sit on and vibrate around the lattice sites. The dimensionality of the lattice can be crucial in certain models, such as the model of magnetic phase transitions. The statistical properties of regular lattices are easy to calculate. The degree distribution of a regular lattice is trivial: P(k) = δkz, where *z* is the coordination number of the lattice. The clustering coefficient depends only on the coordination number for a given dimensionality. For example, for a ring lattice, this number is 0.75(z-2)/(z-1). Obviously, most real networks are not regular lattices, but have intricate structures. The physical distance between the agents may not be very important and very long-range interactions can be present (consider the spread of information on the Internet). The first approximation to such networks is the Random Network model.

**Random Networks**

The first non-regular network model, the random model (also known as the Erdos-Renyi, or ER model), was introduced in the late 1950s [**7**]. To construct such a network, one begins with N isolated nodes, picks pairs of nodes at random, and connects each pair with a constant probability p. This simple construction scheme allows many properties of random networks to be calculated analytically. The mean degree, for example, is simply *<k> = pN*. The degree distribution is a Poissonian:

$$P_{ER}(k)=\frac{{\langle k\rangle }^{k}e^{-\langle k\rangle }}{k!}$$

The average length of the path and of the diameter scales logarithmically with N. The clustering coefficient is CER = p.

The ER model was the only network model in use until the late 1990s, when properties of many man-made and natural networks, such as the World-Wide Web (WWW) or some food webs were investigated. Several studies showed that although certain properties (like average path length) of random networks agree with the empirical data, the degree distributions and clustering coefficients are significantly different. Particularly, the clustering coefficients of most real networks seem to be larger than those of random networks, and independent of the network size [**8**]. The latter is a characteristic of regular lattices. This observation led to the development of another network model.

**Small-world Networks**

Watts and Strogatz [**9**] proposed a new model in 1998 to explain small path lengths and large clustering coefficients independent of the network size — properties shared by many real networks. In this model, one begins constructing a network with a one-dimensional lattice ring of N nodes (or a *d*-dimensional regular lattice), in which each node is wired to its neighbors up to *Kth* nearest neighbor. This regular lattice has a high average path length. To decrease the path length, one rewires each link with a probability *p* to another randomly picked node. This process creates long-range connections. For very small and large values of *p*, a SW network displays characteristics of a regular lattice and an ER network, respectively. Therefore, SW networks lie somewhere between order and randomness. The average path length in SW networks is proportional with N/K weighted with a nonlinear function *f(KN)*. The clustering coefficient for SW networks is proportional with *(1 − p)3*. SW networks share some properties with several real networks. However, their degree distribution, which has a pronounced peak at *<k> = K* and exponentially decaying wings for large K, differ from the power law degree distributions of networks such as the WWW, the Internet, and many social networks.

**Scale-free Networks**

The observation that many real networks have a power-law degree distribution lead Barabasi and Albert to develop yet another model [**7**, **10**]. The model they proposed employs a growth scheme called preferential attachment, in which new nodes are constantly added to the network. However, new nodes are not wired to the existing nodes at random. Rather, each existing node has a probability of making a link to the new one, proportional to its degree. Therefore, high-degree nodes attract more links than others do. This mechanism leads to a power-law degree distribution, *PBA(k) = k−λ*. Subsequent to the Barabasi-Albert (BA) model, several other mechanisms have also been shown to generate power-law degree distributions [**11**].

The average path length in a scale-free network generated by the BA model increases approximately logarithmically with the network size. There are analytical results for the clustering coefficient in certain limits, but usually CBA > CER for networks of comparable size. Moreover, the BA model is known to produce networks with non-trivial degree-degree correlations. Studies of scale-free networks have given us new insights on error and attack tolerance and robustness of various networks [**12**] as well as dynamical processes that take place on networks, such as internet traffic [**13**], spread of infections diseases [**14**], etc.

**Networks and Food Webs**

One of the many applications of network theory is food webs. Food webs are directed networks that depict the “who-eats-whom” relationships in biological communities [**15**]. The vertices in a food-web diagram often do not represent actual species, but trophic species, defined as the sets of species that have the same sets of predators and prey. A directed link from vertex A to vertex B means that species B feeds on species A, indicating the direction of the energy flow. Often, no information about the sizes of the population or the strengths of the interactions is specified. The species with no prey are called the basal species, the species with no predators are called the top species, and all others are called intermediate species. One can use the concept of trophic level to locate a species in a food chain, which is generally defined as the length of the shortest link from the external energy source of that species. Food-web models that do not take into account population dynamics, migration, etc., are called static models, and they employ simple rules to assemble food webs just as in the ER, SW, or BA models mentioned above. For example, the niche model [**16**] builds a web having as properties: the indegree (prey), the outdegree (predator), distributions, their standard deviations, and the fractions of the top and the basal species. 

## Description of the tumor growth process

Tumor growth is a complex process, ultimately dependent on tumor cells proliferating and spreading in host tissues. A very important implication of the spatial and temporal symmetries of tumors is that certain universal quantities can be defined to allow the characterization of the tumor growth dynamics.

Understanding the dynamics of cancer growth is one of the great challenges of modern science. Solid tumors initially develop as a single mass of cells. These divide more rapidly than the cells around them because of a proliferate advantage caused by mutation, and a number of genetic pathways responsible for these mutations have been identified over the last decade [**17**]. 

Because there are three distinct stages (avascular, vascular, and metastatic) to cancer development, researchers often concentrate their efforts on answering specific questions on each of these stages. The avascular stage of tumor growth is characterized by small tumors, which gain the nutrients and oxygen they need for survival and growth by diffusion from external blood vessels. Since there are no blood vessels within the tumor to supply the mass needed for such a volume expansion, this must also enter through the tumor’s periphery. An individual tumor cell has the potential to develop into a cluster of tumor cells, over successive divisions. Further growth and proliferation led to the development of an avascular tumor consisting of approximately 106 cells, which feed on oxygen and other nutrients present in the local environment.

Angiogenesis is the process by which tumors induce blood vessels from the host tissue to sprout capillary tips, which migrate towards and ultimately penetrate the tumor, providing it with a circulating blood supply and, therefore, an almost limitless source of nutrients. 

The vascular growth phase, which follows angiogenesis, is marked by a rapid increase in cell proliferation and is usually accompanied by an increase in the pressure at the centre of the tumor. This may be sufficient to occlude blood vessels and, thereby, to restrict drug delivery to the tumor.

In the earliest stages of development, tumor growth seems to be regulated by direct diffusion of nutrients and wastes from and to the surrounding tissue. When a tumor is very small, every cell receives nourishment by simple diffusion and the growth rate is exponential in time. However, this stage cannot be sustained because the moment a nutrient is consumed its concentration must decrease towards the centre of the tumor. The concentration of a vital nutrient at the centre will fall below a critical level.

After the early stages of growth, the avascular spheroids structurally consist of an inner zone of necrotic cells (dead due to lack of nutrients) and an outer zone of living cells. This outer zone can be further divided into a layer largely composed of quiescent cells and a layer largely composed of proliferating cells, although dead cells are found adjacent to both quiescent and proliferating cells. At this stage, the spheroids tend to reach a finite size of at most a few millimeters in diameter [**18**]. 

However, even in very early stages of their growth, tumors become highly non-homogeneous. Fast growing tumor cells can significantly change their environment leading to formation of gradients of different metabolites, such as oxygen, glucose, growth factors, and other nutrients. Changes in the metabolite concentration cause the development of micro regions occupied by tumor cells of different phenotypes, such as proliferating, quiescent, or necrotic cells. Those subpopulations of cells are characterized by different growth and functional properties as well as by diverse responses to therapeutic factors. 

The biology of tumor micro regions has been investigated experimentally by using the multicell spheroid model [**19**]. In experimental setting, the spheroids are usually initiated from aggregates consisting of several cells, but as their size increases, their growth kinetics becomes similar to those of *in vivo* tumor, such as micro metastases or pre-vascular primary tumors. The multicell spheroids develop a layered structure with a central necrotic core surrounded by quiescent cells and a thin rim of proliferating cells.

## The proposed mathematical model for tumor growth

The process of nutrient consumption and diffusion inside tumors has been modeled since the late 1960’s. Consistent reviews of this area of tumor modeling have been published over the last few years [**20**]. However, they all focus on different aspects of the ones we address. Most models fall into two categories: *continuum mathematical models* that use space averaging and thus consist of partial differential equations and *discrete cell population models* that consider processes on the single cell scale and introduce cell-cell interaction by using cellular automata type of some computational machinery. The model introduced in this paper is based on lattices, and therefore, is a discrete one. 

**Discrete cell population models**

With the huge advances in biotechnology, large amounts of data on phenomena occurring on a single cell scale are now available. This, combined with in vitro experiments using tumor spheroids, sandwich culture, etc., and high power confocal microscopy that enables tracking of individual cells in space and time, has brought about the possibility of modeling single-cell-scale phenomena and then using the techniques of up scaling to obtain information about the large-scale phenomena of tumor growth. There are several up scaling techniques; the most popular ones are cellular automata [**21**], lattice Boltzmann methods [**22**], agent based [**23**], extended Potts [**24**] and the stochastic (Markov chain) approach [**25**]. 

The difficulty with automaton models is the real modeling of cell motion. The first step in setting up rules for cell motion is to partition the physical space into automaton cells. The simplest partition is to discretise into a regular lattice; rectangular lattices are usually chosen due to their simplicity. The second modeling decision is whether the lattice is fixed in time or varies as the elements move. It is far simpler to consider a fixed lattice, with each automaton cell corresponding to either a biological cell or a vacant site, and cells able to move into a nearby lattice site containing a vacant site. In particular, the rules of motion for fixed lattices can be simply formulated in terms of cells moving between lattice sites, if the lattice is free to move and the cells can grow.

**Model implementation**

The basic principles included in the model are cell proliferation, quiescent and necrosis. Each cell has associated with the velocity, which indicates the direction and the distance the cell will move in one time step. There are nine velocity channels in each lattice site: *V0 = (0,0), V1 = (1,-1), V2 = (0,1), V3 = (0,-1), V4 = (-1,-1), V5 = (-1,0), V6 = (-1,1), V7 = (0,1), V8 = (1,1)*, were V0 is the resting channel and *V1, V2, V3, V4, V5, V6, V7 and V8* represent moving to right, up, left, down and diagonals, respectively. In each lattice site, we allow at most one cell (necrotic cells or tumor cells) with each velocity.

Now in each lattice site one of following reactions can occur at each time step:

Quiescent:

{C→i,jCi,jN→i,jNi,j

if and only if (Ci,j ≥ 1)

Proliferation:

{C→i,jC+i,j1N→i,jNi,j

if and only if (Ci,j+Ni,j <5 and Ci,j ≥ 1)

Necrosis:

{C→i,jC−i,j1N→i,jN+i,j1

if and only if (Ci,j ≥ 1)

where C – tumor cells; N – necrotic cells.

In order to address the formation of tumor micro regions, we present a two-dimensional time-dependent mathematical model in which every tumor cell is treated as an individual entity characterized by its own geometry and individually controlled cell processes. This model allows one to follow fate of each individual cell and to investigate how changes occurring in individual cells can influence the behavior of the whole tumor tissue. For simplicity, we introduce only one external metabolic factor in our model and only take into account the effect of nutrient consumption on cell growth and metabolism. 

**Simulation results**

We have developed a computer model based on random growth to simulate the geometrical complexity of a tumor. The model was implemented using Java Development Toolkit.

**Fig. 1:**
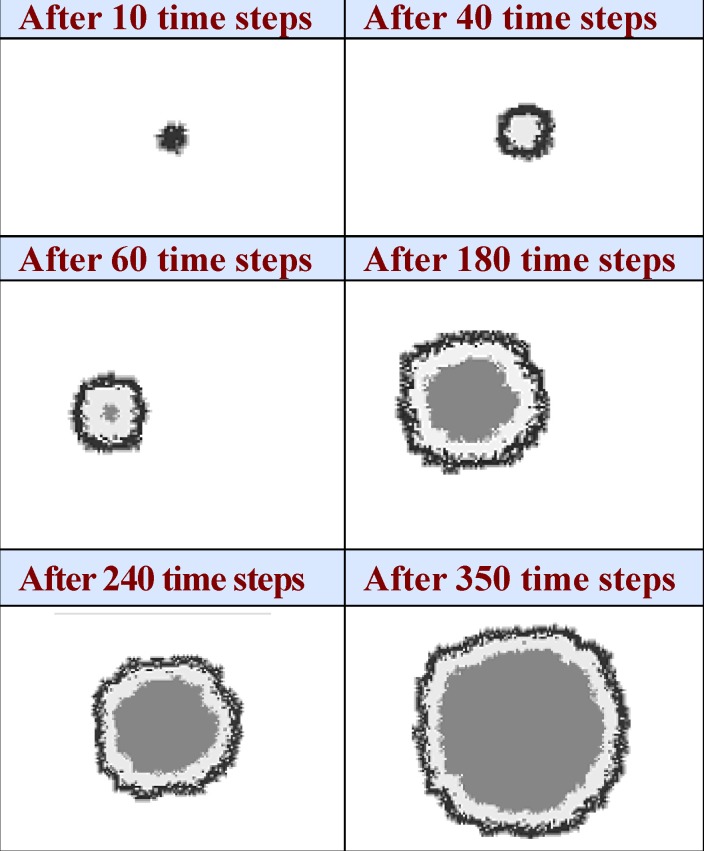
Spatial development of tumor micro regions containing proliferating cells (black), necrotic cells (dark grey), quiescent cells (light grey).

The simulations were performed on a 100 x 100 square lattice with central site initially defined to contain one cancerous cell. The size of the lattice is chosen to be large enough so that the boundaries do not influence the tumor growth within the considered time interval. The simulation has been greatly simplified by neglecting some effects such as: interaction of healthy cells with cancerous cells, the effect of nutrients concentrations and limited volume space for tumor and it seems that the addition of these effects is not problematic in this simulation (**Fig. 1**). 

Our model estimated fast expansion of tumor cells during the first third of the whole period and significant reduction in tumor growth after developing necrotic cell area. Finally, the tumor enters into a phase of growth saturation. The initial fast tumor growth follows from the fact that almost all tumor cells proliferated actively. The percentage of proliferating tumor cells is equal to 100% during this time, except for the scattered single points that reflect short periods of time when the newly created daughter cells did not yet enter in the new cell cycle. A subpopulation of quiescent cells becomes more noticeable at the time the first necrotic cells arise. Once the subpopulation of necrotic cells arises the tumor growth is characterized by a fast exponential expansion. The percentage of the proliferating cells starts to decrease with the increasing of quiescent and necrotic cells.

**Fractal evaluation of the growth process**

By focusing on the irregularity of tumor growth rather than on a single measure of size such as diameter or volume, fractal geometry is well suited to quantify for those morphological characteristics that pathologists have long used in a qualitative sense to describe malignancies – their ragged border with the host tissue and their seemingly random patterns of vascular growth. Herein lays the potential of fractal analysis as a morphometric measure of the irregular structures typical for tumor growth. 

The surface appearance of many malignancies has a typical morphology that corresponds to disease severity: benign tumors are smooth, whereas aggressive malignancies are ‘‘rough’’. There is a correlation between this ‘‘roughness’’ and the tumor’s invasive potential: studies of photomicrographs of tumor surfaces have succeeded in demonstrating self-similarity at different length scales, and have noticed a relationship between the fractal (Hausdorff) dimension of the tumor’s surface and its invasive potential.

**Fig. 2:**
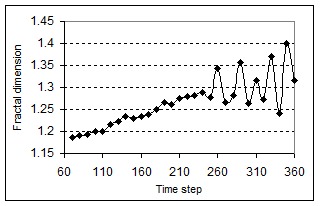
Plot of the fractal dimensions of the tumor boundary as a function of time steps

To measure the fractal dimension and roughness of the tumor boundary we only select the cells at the boundary of the tumors. We define boundary cells as those that have at least one normal neighbor. *Df* was calculated by using a box-counting algorithm [**26**]. See the figure 2 where the fractal dimensions of the patterns are plotted against the total number of time steps. 

Finally, we shall compare the simulated patterns with an *in vitro* model of tumor growth [**27**] for the validation of our computational model.

**Fig. 3:**
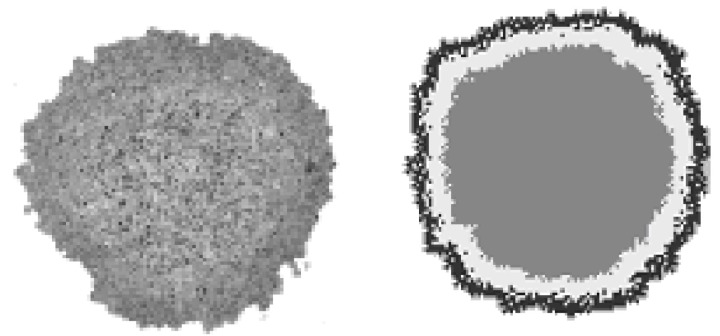
*in vitro* growth model versus bi-dimensional simulation.

In fig. 3 we show two sections of tumor growth: left panel – *in vitro* model and right panel – simulated patterns. This image is very similar to the patterns exhibited in our simulation. In the beginning, we assumed that the similarities between this *in vitro* growth model and the simulated patterns suggested that some of the functional properties of cancer cells were similar to those built in our model.

## Network based growth model with preferential attachment for food webs

 General considerations

Directed networks that transport a resource, such as energy, from one or several sources to a large number of consumers are important in many areas of science. Among such networks, food webs provide an example of great interest, both from a purely scientific point of view and because of their importance for nature-conservation efforts [**28**]. An important aspect of the network structure of food webs is that they have degree distributions that generally decay quite fast with the increasing degree – at least exponentially in most cases [**29**]. This is in sharp contrast with the class of networks known as scale-free, which have power-law degree distributions. While there has been a veritable explosion of research on scale-free networks, there has been no similar surge of interest in networks with rapidly decaying degree distributions. Most food webs, and some (but not all) transportation networks, such as the European railway network belong to this class. As a step towards the development of such an understanding, we here propose a network growth scheme that produces a poissonian indegree distribution (in food-web language: prey distribution) and an outdegree (predator) distribution that is continuously tunable between an exponential distribution and a delta function. We note that these degree distributions do not agree with current food-web theory. In particular, the indegree distribution produced by the niche model has an exponential tail [**16**]. It has been claimed that models with an exponentially decaying probability of preying on a given fraction of species with lower or equal niche values are capable of producing food webs that are structurally in agreement with the empirical data. However, it is not clear why this condition is necessary or why schemes that invoke no physical mechanisms (as in the niche model) are able to produce such webs. In contrast, our model employs a scheme in which new nodes (species) attach to existing nodes with a preference for nodes i with high khi indegree and low *kli* i outdegree. In food-web terms, this corresponds to a prospective predator choosing prey that has a large number of resources (represented by the large indegree), while the competition from the previously established predators should be as small as possible (low outdegree). Among the influences on the growth process of the network mentioned above (speciation, invasion, and extinction), we have thus chosen to focus on invasion and/or speciation, i.e. the early phase of steady network growth. The proposed growth process corresponds to a probability of attachment

$$P(k_{hi},k_{li})={\left(k_{hi}/k_{li}\right)}^{\lambda }$$

(1)

with λ≥0. This attachment scheme is the direct opposite of the preferential attachment scheme, which is known to produce scale-free networks. To emphasize this difference, we shall name the scheme proposed here, inverse preferential attachment. 

**Model and results**

Let us now investigate the general form of the attachment probability presented above. With this form, we relax the restrictions of the simplified model: we do not fix the indegree (number of prey) for the new nodes, so that each can make a different number of links, and we also vary the exponent. This makes full analytical treatment much harder, and the results for the outdegree distribution presented here are therefore only numerical. The indegree distribution, however, is analytically found to be poissonian in the large-network limit.

The generalized attachment process proceeds as it follows. We start the growth process with N0 nodes and assign each initial node an m ≤ N0 indegree. These nodes act as source nodes because they are not connected to any other node at the beginning. Actually, the attributes of the initial nodes have no significance for the statistics because the final size of the network, Nmax + N0, is larger than N0. In addition, we add a new isolated node, each time step. Then, we give the new node m chances to establish a link to an existing probability node

$$P(k_{hi},k_{li})={\left(k_{hi}/(k_{li}+1)\right)}^{\lambda }z^{-1}$$

(2)

where

$$z=\sum_{i=1}^{N}{\left(k_{hi}/(k_{li}+1)\right)}^{\lambda }$$

(3)

with λ≥0. Here, N denotes the number of existing nodes at that time step. We use *kli* + 1 in the denominator to prevent a divergence for *kli* = 0. Multiple links between two nodes are not allowed. The direction of a link is from the old node to the new one.

We implement the growth process in Monte Carlo simulations as it follows. We seed the system with *N0* source nodes, each with m indegree, and introduce a new node in each Monte Carlo step. To create links between the new node and the existing ones, we pick existing nodes, i, one by one and calculate the probabilities of attachment,

$$P(k_{hi},k_{li})$$.

Then, we generate a random number, r, and attach the new node to node i if r < P. We repeat this procedure until all existing nodes in the network are tested.

**Fig. 4:**
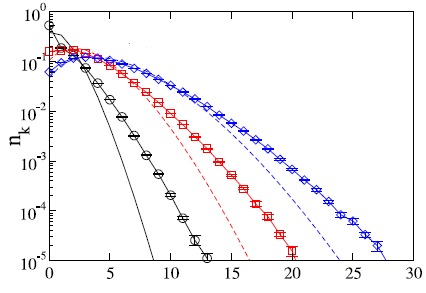
Outdegree distributions for λ=1 and m = 1, 3, and 5 with N0 = 10 shown on a log-linear scale kli(outdegree)

Since

$$\sum_{i=1}^{n}P(k_{hi},k_{li})=1$$

a new node makes on average one connection per sweep. We sweep the whole network m times, so that <khi> = <kli> = m. The new node is kept in the system, even if it does not acquire any links. However, a node with khi = 0 stays isolated throughout the growth since the probability of attachment to it is zero. We stop the growth when the network size N reaches Nmax + N0 nodes with Nmax = 10^5. We average over fifteen independent runs for each value of m and λ. We first tested the case of λ = 1 to compare the outdegree distribution of a full khi/kli model to the outdegree distribution of the simplified 1/ kli model. As seen in (**Fig. 4**), the outdegree distribution for the khi/kli model also decays faster than exponentially for large kli. However, the dependence on the variable indegree leads to a broadening of the outdegree distribution: decreased probabilities for a value of kli around m, and compensating increased probabilities for k greater or less than m. In the limit of large m, the central part of the outdegree distribution of the khi/kli model approaches that of the 1/kli model.

In fig.4 each curve (with symbols) represents an average over fifteen runs. The curves without symbols are the theoretical outdegree distributions for the simplified model. Both the general khi/kli and the simplified 1/kli models yield the same distribution for m >> 1. 

**Fig. 5:**
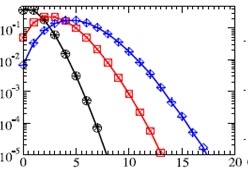
Indegree distributions for λ=1 and m = 1, 3, and 5 with N0 = 10

The outdegree distribution of the general model also varies with λ. Higher values of λ sharpen the peak of the distribution around the mean outdegree, m, as it increases the tendency of the new nodes to prefer existing nodes with a higher value of khi / kli. In the λ→∞ limit one should obtain a delta function at kli = m. Similarly, lower values of λ relax the constraint and flatten the outdegree distribution. The limiting case, kli = 0, corresponds to growth without preferential attachment, which yields an exponential outdegree distribution of mean m. In contrast to the outdegree distribution, the indegree distribution of the generalized model in the N >> m, kli limit can be analytically described and is extremely well approximated by a Poisson distribution with mean m, independent of λ (**Fig. 5**). 

The simulations in fig. 5 were stopped when the network size reached N0 +10^5 nodes. Each curve represents an average over fifteen runs. The symbols *,×, and + show the Poisson distribution for m = 1, 3, and 5, respectively. 

As concluding remarks, let us note that our model produces webs with a positive in-outdegree correlation, whereas the empirical and model webs have a negative correlation [**30**]. This may be due to the unrestrained growth of our networks, which would require an extinction process to achieve a steady state. Moreover, this growth scheme is not designed to produce loops. It would be useful to include such features in future versions of the model, thus enabling modeling of mature, steady-state networks.

## Conclusion

We see the role of mathematical modeling of biological processes as twofold. Models can help our intuition, provide a framework for thinking about the problem, and make predictions. If a model is well parameterized then these predictions can be significant quantitative predictions.

As far as the cancer disease is concerned, mathematical modeling and computer simulations can provide insight into the mechanisms that control tumor evolution and growth, and, hence, suggest directions for new therapies. The theoretical predictions generated from the models and simulations can help optimizing the experimental protocol by identifying the most promising candidates for further clinical investigation. 

We also proposed a new growth scheme for the generation of directed networks, in which new nodes prefer to attach to existing nodes with a lower degree. This model is the opposite of the usual preferential attachment model, which produces scale-free networks. We studied this scheme, not only because it was an unexplored part of the parameter space, but also because we believe that such a mechanism could play an important role in some transportation networks, such as food webs. The scheme we proposed is loosely based on competition between predators and the principle of conservation of energy. The simulation results showed that our networks have degree distributions that fall off at least exponentially for large degrees, like many food webs. However, they have a positive in-outdegree correlation, whereas the empirical and model webs have a negative correlation. We believe that this may be caused by the lack of an extinction mechanism in our model.

Each of the problems we studied can be considered an application of network theory in a broad perspective. Such interdisciplinary problems involving complexity also require a palette of techniques starting with nonlinear dynamics and statistical mechanics. We believe that this body of work, developing around what we call network theory today, will be an important part in the foundation of the twenty-first century complexity science. This will lead to our better understanding of complex phenomena in disciplines ranging from Physics to Biology and the Social Sciences.
